# Case Report: Ductal Carcinoma *In Situ* in the Male Breast

**DOI:** 10.1155/2012/532527

**Published:** 2012-09-26

**Authors:** Joshua Chern, Lydia Liao, Raymond Baraldi, Elizabeth Tinney, Karen Hendershott, Pauline Germaine

**Affiliations:** Departments of Diagnostic Radiology and Surgery, Cooper University Hospital, 1 Cooper Plaza, Suite B23, Camden, NJ 08103, USA

## Abstract

High-grade ductal carcinoma in situ is incredibly rare in male patients. The prognosis for ductal carcinoma in situ (DCIS) in a male patient is the same as it would be for a female with the same stage disease; therefore, early recognition and diagnosis are of the utmost importance. We present a case of a male with unilateral invasive ductal carcinoma who was diagnosed with DCIS in the contralateral breast. The DCIS presented as microcalcifications on mammography and was found to be biopsy proven grade 3 papillary DCIS. This case also illustrates the importance of family history and risk factors, all of which need to be evaluated in any male presenting with a breast mass or nipple discharge.

## 1. Introduction

Ductal carcinoma in situ (DCIS) in males is exceedingly rare. Only a limited number of cases have been reported in the literature. If recognized, early detection of DCIS can have a tremendous impact on mortality. 

## 2. Case Report

A 61-year-old man with an extensive past medical history including hypertension, obesity, noninsulin dependent diabetes mellitus, nonalcoholic steatohepatitis which progressed to cirrhosis, sarcoidosis, and hypothyroidism presented to his family physician with a palpable mass in the right breast. There was no nipple discharge or retraction, however, on physical examination there was bilateral symmetric gynecomastia as well as inflammatory changes of the skin of the right breast. 

This gentleman has an extensive family history of cancer. His sister died at age 58 of metastatic breast cancer and his father succumbed to complications from melanoma. Additionally, he has a niece who was recently diagnosed with breast cancer.

**Figure 1:**
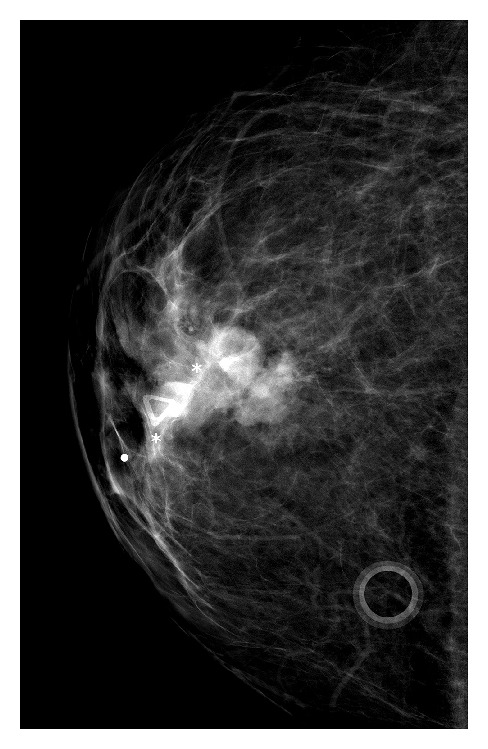
Right craniocaudal image demonstrates a highly suspicious retroareolar mass which correlates to the palpable abnormality.

**Figure 2:**
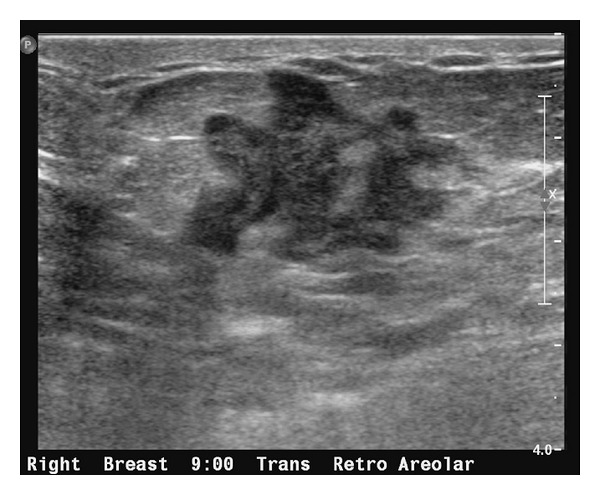
Focused ultrasound of the right breast in the retroareolar region better demonstrates the biopsy-proven invasive breast cancer.

**Figure 3:**
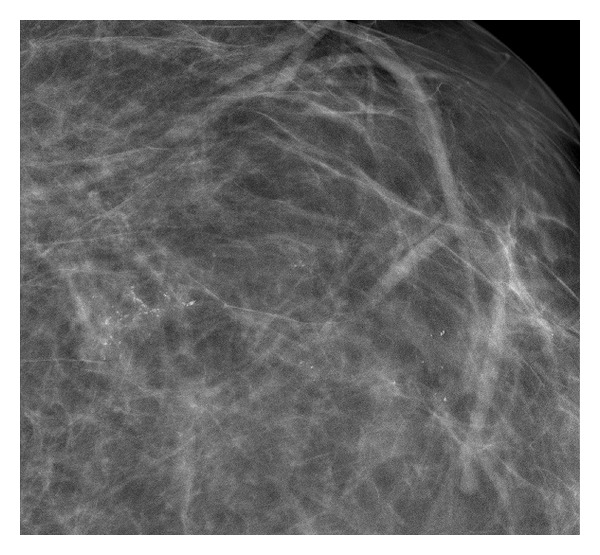
Left magnified craniocaudal image shows branching pleomorphic calcifications.

This patient was referred for bilateral diagnostic mammograms and targeted high-resolution ultrasound of the right breast mass (Figures 1, 2, and 3). The mammogram showed a suspicious retroareolar mass in the right breast and two foci of pleomorphic microcalcifications within the left breast. The patient returned 3 days later for a stereotactic biopsy of the microcalcifications in the left breast and an ultrasound guided biopsy of the right breast mass. The pathology revealed grade 2-3 invasive ductal carcinoma of the right breast and ductal carcinoma in situ of the left breast. 

Subsequently, a simple mastectomy was performed on the left and a modified radical mastectomy was performed on the right. Sentinel lymph nodes were biopsied bilaterally. No nodal metastatic disease was identified. The lesions were found to be estrogen/progesterone receptor positive bilaterally, however, interestingly only the left breast DCIS was HER2 positive. The DCIS was grade 3 and its pathologic subtype was focal and solid pappilary.

The patient underwent subsequent genetic testing which was negative. His surgery resulted in a left chest wall hematoma, but was otherwise uneventful. To date, the patient remains cancer free.

## 3. Discussion

Ductal carcinoma in situ in the male breast is exceedingly rare. There are very few cases of pure DCIS in the literature to date. Generally, male breast cancer presents as an invasive carcinoma. Our case is highly unusual in that the patient presented with invasive carcinoma and was discovered to have coexisting DCIS in the contralateral breast.

Compared to its female counterpart, DCIS in males tends to present at a more advanced stage and at a later age [1]. It tends to present as a retroareolar, palpable mass, and in many cases bloody nipple discharge [1, 2]. 

The most common dominant pathologic subtypes of DCIS in males are papillary and cribriform, respectively [2, 3]. It has been shown that invasive breast cancers in males are less likely to be of the papillary subtype. These findings suggest that papillary lesions are more likely to expand and remain in situ than other subtypes. In the largest study to date regarding male DCIS, there were 0 cases of pure DCIS presenting with grade 3 features [2]. Our case is unusual in this matter. The primary pathologic subtype of DCIS in our patient was papillary, however, it was grade 3. 

In contrast to the female breast, the male breast generally only develops the more central ducts. Lobules, or the terminal ductal lobular units (TDLU), are only developed in the presence of estrogen or estrogen-like compounds. It is felt that higher-grade DCIS is derived from the epithelial cells in the TDLU. These cells are only present in male breasts in patients with hyperestrogenism [2]. Our patient has cirrhosis and obesity that could result in an elevated estrogen level. Other factors that could predispose a male to breast carcinoma would be Klinefelter's syndrome, hormone therapy for prostate carcinoma, and gynecomastia [1, 2]. Family history is another important factor that predisposes men to breast cancer and DCIS. There have been associations with the BRCA2 gene mutation in many cases of male breast carcinoma [3]. Surprisingly, given our patient's extensive family history, he did test negative for BRCA1 and 2.

There is no difference in prognosis between male and female breast carcinoma when compared at similar stages and thus, it is important to recognize male breast carcinoma in its earlier stages. In both sexes, DCIS has a favorable prognosis, which is why any male with nipple discharge should prompt further investigation. This especially holds true in men with some of the predisposing factors mentioned above, as in our case.
